# Consistent predictors of adolescent substance use: cross-sectional comparison across five countries

**DOI:** 10.3389/fpsyg.2026.1737472

**Published:** 2026-03-12

**Authors:** Kourosh Bador, Anis Sfendla, Hang T. M. Nguyen, Catrin Johansson, Nóra Kerekes

**Affiliations:** 1 Centre for Holistic Psychiatry Research (CHoPy), Mölndal, Sweden; 2 AGERA KBT, Gothenburg, Sweden; 3 High Institute of Nursing Professions and Health Techniques, Fez, Morocco; 4 Faculty of Psychology, University of Social Sciences and Humanities, Hanoi, Vietnam; 5 Department of Health Sciences, University West, Trollhättan, Sweden

**Keywords:** adverse childhood experiences, alcohol use, Big Five personality, cross-cultural comparison, drug use, psychological distress

## Abstract

**Background:**

Adolescent substance use differs across cultures, but the key drivers may be shared. We examined whether country of residence, psychological distress, adverse childhood experiences (ACEs), and Big Five personality traits are associated with alcohol and drug use, and whether these associations vary by country.

**Methods:**

We conducted a cross-sectional online survey of 5,108 upper-secondary students aged 15–19 from the United States, Sweden, Serbia, Morocco, and Vietnam. Outcomes were scores on the Alcohol Use Disorders Identification Test (AUDIT) and the Drug Use Disorders Identification Test (DUDIT). Predictors included country, age, gender, psychological distress, four ACEs (family alcohol problems, family drug problems, physical abuse, psychological abuse), and Big Five traits.

**Results:**

Alcohol use was higher in Sweden and Serbia and lower in Morocco and Vietnam compared with the United States. Drug use was highest in the United States and lower in all other countries (lowest in Vietnam and Serbia). Older age predicted higher AUDIT and DUDIT scores. Gender was unrelated to alcohol use, whereas drug use was highest among young men. Psychological distress showed the strongest positive association with both outcomes. For alcohol, family alcohol and family drug problems predicted higher use; physical and psychological abuse were not significant. For drugs, family drug problems and experiences of physical and psychological abuse predicted higher use; family alcohol problems did not. Personality effects were consistent: extraversion predicted higher use, whereas conscientiousness, agreeableness, openness, and—more modestly for alcohol—neuroticism predicted lower use. Associations were largely stable across countries.

**Conclusion:**

While levels of alcohol and drug use differ across countries, the core predictors are consistent: psychological distress and early adversity elevate risk, whereas conscientiousness and agreeableness are protective and extraversion signals vulnerability. Prevention efforts should combine trauma-informed, mental-health approaches with personality-targeted programs. Limitations include uneven country distributions and pandemic timing.

## 1 Introduction

Substance use, including alcohol and drug consumption, is a critical public health issue that contributes to increased risks of morbidity, mortality, and social consequences (World Health Organization, 2024). Adolescence is a particularly vulnerable developmental period for substance use initiation, as rapid biological, psychological, and social changes increase susceptibility to risky behaviors (Kuhn et al., 2013). This developmental period is characterized by increased autonomy, shifting social influences, and heightened risk-taking tendencies, which contribute to the likelihood of substance use initiation (Giedd, 2015; Spear, 2000). Studies have shown that older adolescents are more likely to engage in substance use than younger adolescents partly due to greater exposure to peer influence and increased access to alcohol and drugs (Steinberg, 2008).

However, the extent to which adolescents engage in alcohol use varies significantly across cultures, reflecting differences in societal norms, legal regulations, and accessibility of substances (Sudhinaraset et al., 2016). In addition, the extent to which age-related patterns in substance use vary across cultures remains an important area of study, as societal norms and policy restrictions shape how adolescents of different ages engage with substances. Understanding these cultural variations is essential for developing effective, context-specific prevention and intervention strategies.

Numerous studies have demonstrated age-related patterns in adolescent substance use vary across cultures, influenced by social norms and policy restrictions. For example, research has found that alcohol consumption is more prevalent in American and European countries, where drinking is often integrated into social and family life (Sudhinaraset et al., 2016; Sfendla et al., 2022). By contrast, countries with religious or legal restrictions —where alcohol consumption is prohibited in Islam and officially banned in most Muslim-majority nations—report the lowest incidence of alcohol use globally (World Health Organization, 2011). This pattern is also evident among adolescents, with studies showing that youth in countries with Islamic cultural influences tend to report particularly low rates of drinking (Kuntsche et al., 2014; Unlu and Sahin, 2016). Similarly, Buddhist beliefs may discourage alcohol consumption; however, findings on actual drinking patterns among Buddhists vary across contexts. While some studies suggest lower alcohol consumption among Buddhists compared to others within the same cultural context (Newman et al., 2006), others have reported higher prevalence rates in certain populations (Kumbukage et al., 2025). Additionally, Vietnamese children often live in multigenerational families (e.g., with great grandparents, grandparents, and parents living together) in which their behaviors outside school hours are closely supervised (Jordan et al., 2013), that may have been the specific situation for many adolescents as well during the COVID-restrictions (Nguyen, 2023).

Similar trends are observed in drug use, as cultural and policy-related factors strongly influence adolescent behavior. Countries with strict drug laws and severe legal consequences, such as Vietnam and Morocco, report low levels of adolescent drug use, while nations with high substance availability and more permissive attitudes, such as the United States, tend to report high levels of adolescent drug consumption (Johnston et al., 2022). These cultural variations suggest that substance use behaviors are not only shaped by individual choices but also by broader societal structures and norms.

Beyond cultural influences, psychological distress and adverse childhood experiences (ACEs) consistently increase the risk of substance use across diverse populations (Ajith et al., 2025). Psychological distress—including anxiety, depression, and emotional dysregulation—is strongly linked to both alcohol and drug use, as many adolescents use substances as a coping mechanism (Leza et al., 2021). This association appears largely universal, as studies across high-, middle-, and low-income countries have found that psychological distress significantly increases adolescent substance use regardless of cultural context (Tian et al., 2021).

Similarly, ACEs such as family substance use, physical abuse, and psychological abuse are strongly associated with increased alcohol and drug use (Felitti et al., 1998; Dube et al., 2003; Sebalo et al., 2023). Childhood trauma has been shown to alter emotional regulation, increase impulsivity, and psychological distress (Johansson et al., 2024) and heighten vulnerability to peer pressure (Giletta et al., 2021), leading to greater engagement in substance use (Lee and Chen, 2017; Broekhof et al., 2023). Importantly, research suggests that the impacts of ACEs on substance use are stable across cultures, reinforcing the need for early intervention strategies that address childhood trauma as a universal risk factor (Kessler et al., 2010).

While cultural context plays a dominant role in shaping adolescent substance use, individual personality traits also contribute to risky behaviors. In the Big Five literature, extraversion is associated with increased risk for alcohol and drug use, whereas conscientiousness, agreeableness, and neuroticism have been associated with lower substance use (Lui et al., 2022). Personality associations appear largely universal influence on substance use, with extraversion increasing risk and conscientiousness providing protection regardless of cultural background (Mezquita et al., 2018; Kuntsche et al., 2017).

Given the complex interplay between cultural influences, psychosocial factors, and individual characteristics in shaping adolescent substance use, this study examined substance use predictors across five culturally diverse countries: the United States, Sweden, Serbia, Morocco, and Vietnam. Specifically, we investigated whether cultural background, psychological distress, ACEs, and personality traits predict alcohol and drug use among adolescents; whether substance use patterns differ across cultural contexts; and whether the effects of key psychological and demographic predictors on substance use vary by country. These countries were selected based on an established international collaboration and were chosen to reflect a broad range of cultural, economic, and policy contexts related to adolescent substance use.

By identifying both universal and culture-specific influences, this study contributes to a better understanding of adolescent substance use behaviors across diverse populations. These insights can inform culturally tailored prevention strategies and intervention efforts aimed at reducing substance-related harm among adolescents worldwide.

Based on previous research, we hypothesize that higher extraversion is associated with increased substance use and that higher conscientiousness, agreeableness, and neuroticism are associated with lower substance use across all cultural contexts. Additionally, psychological distress and the presence of ACEs are expected to be associated with higher substance use, which is also expected to be dependent on respondents’ age and gender.

Given the multinational nature of the study, we hypothesize that substance use patterns differ across countries, reflecting cultural variations in alcohol and drug consumption behaviors.

By testing these hypotheses, this study aims to provide a comprehensive understanding of the psychological and cultural factors that influence substance use. The findings will inform culturally sensitive prevention and intervention strategies, ensuring more effective and tailored approaches to addressing substance use behaviors in diverse populations.

## 2 Subjects and methods

### 2.1 Procedure

Within the frame of the international Mental and Somatic Health without borders (MeSHe) project,^1^ data were collected from upper secondary school students between September 2020 and February 2021 using an electronic survey. A detailed description of the data collection process can be found in Kerekes et al. (2021). The MeSHe survey, which was made available in the participating countries’ languages, consists of several validated questionnaires that capture information about adolescents’ self-rated mental and physical health, risk behaviors, levels of leisure time and physical activity, general affects, and personalities. All instruments used had existing validated versions in each language. No additional translation was conducted by the research team for this study.

### 2.2 Participants

The analyses included 5,108 total responses from individuals aged 15–19 years from the five participating countries. Of these respondents, 0.5% (*n* = 23) did not indicate their gender, while the remaining participants (*n* = 5,085) were categorized as young women (61.6%, *n* = 3,145), young men (36.8%, *n* = 1,882), or non-binary adolescents (1.1%, *n* = 57). The distribution of data was not equal across the participating countries: Sweden accounted for 31.4% (*n* = 1,606) of the responses, Vietnam accounted for 30% (*n* = 1,532), Serbia accounted for 21.7% (*n* = 1,107), Morocco accounted for 10.6% (*n* = 540), and the United States accounted for 6.3% (*n* = 323).

The response rates for the Big Five Inventory (BFI) scales were between 86.9 and 87.6%; for the Alcohol Use Disorders Identification Test (AUDIT), 77.1%; and for the Drug Use Disorders Identification Test (DUDIT), 76.4%.

### 2.3 Instruments

#### 2.3.1 Demographic variables

Country of residence, age, and gender (young man, young woman, or non-binary) were self-reported by the adolescents.

#### 2.3.2 Drug use disorders identification test

The DUDIT contains 11 questions that capture the frequency of an individual’s drug use during the prior year using self-reports. Items 1–9 are each rated on a 5-point Likert scale, while items 10 and 11 are each rated on a 3-point Likert scale. The DUDIT has previously been used in adolescent populations (Liskola et al., 2021). The Cronbach’s alpha for the DUDIT in the present study was 0.94, reflecting the high internal-reliability of the instrument in this sample.

#### 2.3.3 Alcohol use disorders identification test

In 1993, the World Health Organization developed the AUDIT (Saunders et al., 1993), which was intended to identify early hazardous and harmful drinking. The instrument contains 10 items. The first eight items are each scored on a 5-point Likert scale; the last two items are each scored as 0, 2, or 4 points; the total score ranges from 0 to 40. Items 1–3 reflect the individual’s consumption score, and items 4–10 reflect the problem score (Kuitunen-Paul et al., 2018). Three of the items focus on alcohol use, four on dependence, and three on problems related to alcohol habits. The AUDIT has previously been used to assess alcohol problems among the general population (Aalto et al., 2009) and adolescents (Martin et al., 2014; Rumpf et al., 2013; Sfendla et al., 2022). The Cronbach’s alpha for the AUDIT in the present study was 0.78, indicates acceptable internal reliability in this sample.

#### 2.3.4 Big Five inventory

The BFI comprises 44 short-phrase items that measure five factors: extraversion (8 items), agreeableness (9 items), conscientiousness (9 items), neuroticism (8 items), and openness (10 items). Participants are asked to express their degree of agreement or disagreement with each item using a 5-point Likert scale, with the options ranging from “strongly disagree” to “strongly agree” (John and Srivastava, 1999). In this study, the Cronbach’s alpha for these factors ranged from 0.75 to 0.80.

#### 2.3.5 Brief symptom inventory

The Brief Symptom Inventory (BSI; Derogatis and Melisaratos, 1983) is a multidimensional assessment tool used to evaluate mental health status. It comprises 53 items that measure somatic, emotional, and behavioral symptoms. Participants rated the intensity of their distress for each item over the past 7 days using a 5-point Likert scale ranging from 0 (not at all) to 4 (extremely). Item scores were summed to generate domain scores, which were then averaged to calculate the Global Severity Index (GSI), a measure of overall psychological distress. The Cronbach’s alpha for GSI was 0.93.

#### 2.3.6 Questions regarding adverse childhood events (ACEs) and trauma

The background questionnaire section of the MeSHe survey contained four questions related to ACEs. Participants were asked to respond to these items with a simple “yes” or “no.” The questions were presented as follows:

1. Do any of the adults you live with have a problem with alcohol (alcoholism)?
2. Do any of the adults you live with have a problem with drugs?
3. Have you ever experienced physical abuse (for example, have you been pushed, kicked, beaten, or slapped)?
4. Have you ever experienced psychological abuse (for example, have you been threatened, forced to do something that feels wrong, or violated by humiliating and insulting words)?

### 2.4 Statistical analysis

Because AUDIT and DUDIT total scores were positively skewed with many zeros and clear overdispersion (variance ≫ mean), we used negative binomial regression with a log link to model each outcome. Two models were estimated separately with AUDIT (alcohol-use severity) and DUDIT (drug-use severity) as dependent variables. Predictors were specified as factors (categorical: gender, country of residence, family alcohol problems, family drug problems, physical abuse, psychological abuse) and covariates (continuous: Big Five personality traits [OCEAN] and psychological distress [BSI-GSI]).

The main (full) model for each outcome included all predictors simultaneously to estimate their independent associations. We report log-scale coefficients (*β*) and standard errors (SE) together with incidence rate ratios (IRR) calculated as IRR = exp.(*β*) and 95% confidence intervals exp.(*β* ± 1.96 × SE). Because no exposure offset was used, the IRR is interpreted as the multiplicative change in the expected AUDIT/DUDIT score associated with a one-unit increase in a continuous predictor (e.g., BSI-GSI or a Big Five trait) or, for categorical predictors, relative to the reference category, adjusting for the other variables. For example, IRR = 1.20 indicates a 20% higher expected score; IRR = 0.80 indicates a 20% lower expected score.

To test cross-national moderation, we estimated separate interaction models in which country was interacted one-at-a-time with each predictor that was significant in the corresponding main model; this approach minimized multicollinearity and convergence issues. Statistical significance for main effects and interactions was evaluated using Type III likelihood-ratio χ^2^ tests. Overall model adequacy was assessed with likelihood-ratio tests, and two-sided *p*-values with *α* = 0.05 were used throughout.

All analyses were performed using SPSS version 30 (IBM).

## 3 Results

### 3.1 Alcohol use predictors in a multinational sample

The regression analysis examined the association between AUDIT scores (which measured alcohol use behavior) and demographic predictors (country of residence, age, and gender), psychological distress, specific ACEs, physical disabilities and personality traits (Table 1).

**Table 1:** Results of the negative binomial regression main effect model for AUDIT scores.

Predictor variable	*β* coefficient	Exp(*β*) (IRR)	SE	95% CI	*p*-value
Intercept	−3.553	0.029	0.6051	[−4.739, −2.367]	<0.001
Country of residence (Ref: “USA”)					
Sweden	0.517	1.677	0.1123	[0.297, 0.737]	<0.001
Morocco	−1.694	0.184	0.1669	[−2.021, −1.367]	<0.001
Serbia	0.707	2.028	0.1175	[0.477, 0.937]	<0.001
Vietnam	−0.456	0.634	0.1195	[−0.691, −0.222]	<0.001
Age	0.293	1.341	0.0259	[0.243, 0.344]	<0.001
Gender (Ref: “Non-binary”)					
Young men	0.073	1.076	0.2185	[−0.355, 0.501]	0.74
Young women	−0.251	0.778	0.2169	[−0.677, 0.174]	0.25
Psychological distress (GSI)	0.561	1.752	0.0456	[0.472, 0.650]	<0.001
Family alcohol problem (Ref: “Yes”)	−0.285	0.752	0.0818	[−0.445, −0.125]	<0.001
Family drug problem (Ref: “Yes”)	−0.389	0.678	0.1861	[−0.753, −0.024]	0.037
Physical abuse (Ref: “Yes”)	−0.075	0.928	0.0617	[−0.196, 0.046]	0.23
Psychological abuse (Ref: “Yes”)	−0.011	0.989	0.0591	[−0.127, 0.105]	0.90
Big Five personality traits					
Openness	−0.139	0.870	0.0392	[−0.216, −0.062]	<0.001
Conscientiousness	−0.203	0.817	0.0413	[−0.284, −0.122]	<0.001
Extraversion	0.463	1.589	0.0384	[0.388, 0.538]	<0.001
Agreeableness	−0.257	0.773	0.0529	[−0.361, −0.153]	<0.001
Neuroticism	−0.090	0.914	0.0442	[−0.176, −0.003]	0.043

IRR, incidence rate ratio; CI, confidence interval; SE, standard error.

Country of residence played a key role, with participants from Sweden (IRR = 1.677, *p* < 0.001) and Serbia (IRR = 2.028, *p* < 0.001) reporting significantly higher AUDIT scores compared to those from the United States, suggesting greater alcohol use. By contrast, individuals from Morocco (IRR = 0.184, *p* < 0.001) and Vietnam (IRR = 0.634, *p* < 0.001) had significantly lower AUDIT scores compared to those from the United States.

Age emerged as a significant predictor (IRR = 1.341, *p* < 0.001), indicating that alcohol use tends to increase with age. Gender was not a significant predictor of alcohol use.

Psychological distress had the strongest effect on alcohol use (IRR = 1.752, *p* < 0.001), suggesting that individuals experiencing higher levels of psychological distress reported significantly greater alcohol consumption.

Furthermore, of the ACEs, family history of substance use problems was associated with alcohol consumption. Individuals without a family history of alcohol problems had lower AUDIT scores (IRR = 0.752, *p* < 0.001), as did those without a family history of drug problems (IRR = 0.678, *p* = 0.037).

Personality traits were also found to be important for understanding alcohol use behaviors. Higher extraversion was associated with increased AUDIT scores (IRR = 1.589, *p* < 0.001), suggesting that more extraverted individuals tend to consume more alcohol. By contrast, higher levels of conscientiousness (IRR = 0.817, *p* < 0.001), openness (IRR = 0.870, *p* < 0.001), agreeableness (IRR = 0.773, *p* < 0.001), and neuroticism (IRR = 0.914, *p* = 0.043) were all linked to lower AUDIT scores, indicating that individuals with these personality traits tend to drink less.

In the next step, the interaction between country of residence and significant predictors from the main effect model—age, psychological distress, family history of alcohol and drug problems, and personality traits—were examined on AUDIT scores. None of the interaction effects were significant, suggesting that although these predictors have a direct impact on AUDIT scores, their effects do not depend on cultural differences.

### 3.2 Drug use predictors in a multinational sample

The results of the regression analysis of the predictors of DUDIT scores, which assess drug use behaviors, suggest that all the included factors were found to be significantly associated with drug use (Table 2).

**Table 2:** Results of the negative binomial regression main effect model for DUDIT scores.

Predictor variable	*β* coefficient	Exp(*β*) (IRR)	SE	95% CI	*p*-value
Intercept	0.737	2.089	0.813	[−0.856, 2.330]	0.37
Country of residence (Ref: “USA”)					
Sweden	−1.648	0.192	0.117	[−1.878, −1.419]	<0.001
Morocco	−1.839	0.159	0.155	[−2.142, −1.536]	<0.001
Serbia	−2.076	0.125	0.134	[−2.339, −1.813]	<0.001
Vietnam	−2.378	0.093	0.140	[−2.652, −2.104]	<0.001
Age	0.240	1.271	0.036	[0.169, 0.311]	<0.001
Gender (Ref: “Non-binary”)					
Young men	1.341	3.824	0.310	[0.733, 1.949]	<0.001
Young women	0.375	1.455	0.309	[−0.231, 0.980]	0.23
Psychological distress (BSI_GSI)	1.106	3.022	0.063	[0.982, 1.230]	<0.001
Family alcohol problem (Ref: “Yes”)	0.166	1.181	0.116	[−0.062, 0.395]	0.15
Family drug problem (Ref: “Yes”)	−0.846	0.429	0.186	[−1.209, −0.482]	<0.001
Physical abuse (Ref: “Yes”)	−0.214	0.807	0.085	[−0.381, −0.047]	0.012
Psychological abuse (Ref: “Yes”)	−0.289	0.749	0.082	[−0.451, −0.128]	<0.001
Big Five personality traits					
Openness (BFI_O)	−0.161	0.851	0.059	[−0.277, −0.046]	0.006
Conscientiousness (BFI_C)	−0.517	0.596	0.059	[−0.633, −0.401]	<0.001
Extraversion (BFI_E)	0.318	1.375	0.053	[0.214, 0.423]	<0.001
Agreeableness (BFI_A)	−0.452	0.636	0.073	[−0.594, −0.310]	<0.001
Neuroticism (BFI_N)	−0.501	0.606	0.063	[−0.624, −0.377]	<0.001

Country of residence was a strong predictor of drug use, with participants from Sweden, Morocco, Serbia, and Vietnam reporting significantly lower DUDIT scores compared to those from the United States. Specifically, individuals from Vietnam (IRR = 0.093, *p* < 0.001) and Serbia (IRR = 0.125, *p* < 0.001) had the lowest predicted drug use, followed by Morocco (IRR = 0.159, *p* < 0.001) and Sweden (IRR = 0.192, *p* < 0.001).

Gender was a significant predictor, with adolescent men reporting significantly higher DUDIT scores compared to individuals who identified as neither men nor women (IRR = 3.824, *p* < 0.001). However, the difference between women and the reference group was not statistically significant (*p* = 0.23).

Age was a significant predictor of drug use, with older individuals reporting higher DUDIT scores (IRR = 1.271, *p* < 0.001). This suggests that drug use increased with age in this sample, with each additional year being associated with a 27.1% increase in expected drug use scores.

As with alcohol use, psychological distress had the strongest effect on drug use, with individuals reporting higher distress showing significantly greater drug use (IRR = 3.022, *p* < 0.001), suggesting that psychological distress is a key driver of drug use behavior.

Regarding ACEs, adolescents without a family history of drug problems had lower DUDIT scores (IRR = 0.429, *p* < 0.001). Experiencing physical or psychological abuse was significantly associated with higher drug use, as participants who had not experienced physical abuse (IRR = 0.807, *p* = 0.012) or psychological abuse (IRR = 0.749, *p* < 0.001) had lower DUDIT scores. In other words, these three ACEs significantly predicted increased drug use.

Extraversion was associated with higher drug use (IRR = 1.375, *p* < 0.001), while openness (IRR = 0.851, *p* = 0.006), conscientiousness (IRR = 0.596, *p* < 0.001), agreeableness (IRR = 0.636, *p* < 0.001), and neuroticism (IRR = 0.606, *p* < 0.001) were all significantly associated with lower DUDIT scores.

To examine whether the relationships between significant predictors of drug use varied across cultural contexts, we conducted a series of interaction analyses. The results revealed several significant overall interactions, suggesting potential cross-cultural differences in how certain factors influence drug use. However, post-hoc analyses did not consistently identify significant country-specific differences, indicating that while cultural variation may exist, it is not always strong enough to reach statistical significance at the individual country level.

The interaction between country and age was significant (*p* = 0.048), suggesting that the association between age and drug use differs across cultures. However, none of the individual country-level interactions reached statistical significance, meaning that while age-related patterns in drug use may vary internationally, no single country exhibited a distinctly different trend.

Similarly, psychological distress was consistently associated with higher drug use across all countries (Exp(B) = 1.854, *p* < 0.001), but the strength of this relationship varied slightly between cultural contexts. Serbia exhibited a close to significant interaction (*p* = 0.06), suggesting that the effect of psychological distress on drug use may be stronger in Serbia than in the other countries studied.

The findings also suggest that the impact of ACEs on drug use varies across cultural contexts. Significant interactions (*p* < 0.001) indicated that the effects of physical and psychological abuse on drug use differ between countries. Additionally, the influence of family drug problems on substance use was particularly pronounced in Serbia, highlighting cross-cultural differences in how familial substance use shapes individual risk. However, as with other interactions, specific country comparisons did not consistently reach statistical significance.

Finally, two interactions were not significant. First, the interactions between country and personality traits (OCEAN) were not statistically significant, indicating that the associations between personality traits and drug use were consistent across cultures. For example, conscientiousness, typically linked to lower substance use, and extraversion, often associated with higher use, showed similar effects in all examined countries. Second, the interaction between country and gender did not reveal any statistically significant effects, suggesting that gender-related patterns in drug use remain stable across cultural contexts.

## 4 Discussion

This study investigated how personality traits, psychological distress, ACEs, and demographic factors relate to adolescent alcohol and drug use across five culturally diverse countries. While prevalence rates differed by country, the core predictors of substance use showed notable consistency. These findings support the idea that certain psychological and developmental risk factors operate similarly across diverse sociocultural settings.

### 4.1 Cultural differences and the role of age and gender in substance use

Our findings confirmed significant cross-cultural variations in both alcohol and drug use, aligning with previous research highlighting the role of cultural norms, policies, and societal attitudes in shaping adolescent substance use behaviors (Sudhinaraset et al., 2016; Sfendla et al., 2022; Kuntsche et al., 2014; Unlu and Sahin, 2016; Newman et al., 2006).

Alcohol use was highest among adolescents in Sweden and Serbia, exceeding reported levels in the United States, while Morocco and Vietnam had significantly lower AUDIT scores. These differences may reflect cultural norms around alcohol consumption, with Sweden and Serbia having more permissive drinking cultures or social environments where alcohol consumption is normalized during adolescence. Prior research suggests that in some cultures, alcohol is integrated into adult socialization, which may influence adolescent drinking behaviors as they transition into adulthood (Sfendla et al., 2022). By contrast, Morocco and Vietnam, which have stronger cultural or religious restrictions on alcohol, and also had periods of complete locked downs during the COVID when stronger parental controls could be expected (Nguyen, 2023), showed notably lower consumption rates.

Drug use was lowest in Vietnam and Serbia and highest in the United States, which is consistent with previous reports on problematic adolescent drug use in the United States (ESPAD, 2016). Factors such as greater accessibility, differences in drug policies, and varying levels of enforcement may contribute to these discrepancies. Additionally, the stigma surrounding drug use differs across cultures, potentially influencing self-reported behaviors (ESPAD, 2016). The particularly low drug use in Vietnam and Serbia, as described in the introduction, might be explained by strict drug laws, cultural disapproval, or limited availability, whereas the higher prevalence in the United States could reflect broader societal trends related to substance accessibility and use.

Besides cultural variations, our findings also highlight age as a significant predictor of both alcohol and drug use, with older adolescents reporting higher AUDIT and DUDIT scores. This pattern is consistent with previous research suggesting that as adolescents age, they gain greater autonomy, increased social exposure, and more opportunities to engage in substance use (Steinberg, 2008). Peer influence is particularly relevant, as older adolescents are more likely to socialize in environments where alcohol and drugs are present, increasing their likelihood of experimentation and regular use (Watts et al., 2024). Additionally, age-related changes in risk perception may contribute to these trends, as younger adolescents aged ten to fourteen tend to have stronger parental supervision and greater sensitivity to potential risks, whereas older adolescents aged fifteen to nineteen may perceive substance use as less harmful or more socially acceptable.

Adolescent men reported significantly higher drug use than individuals identifying as neither men nor women; however, no significant gender differences were observed for alcohol use. Similar results were previously reported among a sample of Swedish adolescents (Sfendla et al., 2022).

These findings, along with prior research (ESPAD, 2016; Amaro et al., 2001), suggest that socialization processes and societal expectations may narrow the gender gap in substance use, particularly in alcohol use. In many cultures, alcohol consumption is widely accepted for both men and women, especially in social settings, which could explain the lack of significant gender differences in AUDIT scores.

While our findings may appear to contrast with studies reporting strong gender differences in alcohol use among adults—such as Kumar et al. (2023), who found markedly higher drinking rates among Vietnamese men—these differences likely reflect age-related shifts in social roles, access, and cultural expectations. Similar patterns are observed in other countries represented in our study. For example, in Sweden, adult men consistently report higher levels of alcohol consumption than women (Guttormsson, 2025); in Morocco, cultural and religious norms strongly discourage alcohol use among women, resulting in gendered drinking patterns among adults (Ben El Jilali et al., 2020; El Omari et al., 2015). In the United States, adult gender differences persist but have narrowed over time (Keyes et al., 2021) while Serbian data also suggest higher rates of alcohol use among adult men (Mihailovic et al., 2020). These differences further highlight the importance of age and context in interpreting gendered substance use patterns. Our school-based adolescent sample represents a younger population where such patterns may not yet be fully established.

Studies suggest that young men are generally more exposed to peer pressure related to drug use, more likely to engage in risk-taking behaviors, and may have greater access to illicit substances (Studer et al., 2016; United Nations Office on Drugs and Crime, 2021). By contrast, girls often initiate drug use as coping strategies to manage depression, anxiety, and other negative emotions (Chen et al., 2004). These divergent patterns underscore how gendered social norms and psychological stressors shape distinct pathways into substance use (Chen et al., 2004; Khan et al., 2013; McHugh et al., 2018).

Additionally, cultural attitudes toward women’s substance use may discourage young women from seeking treatment or reporting their use honestly in surveys. Beyond stigma, fears of family separation and punitive legal measures further deter women from disclosing substance use or accessing care, contributing to the observed gender disparity in drug use data. These dynamics reflect deeply gendered expectations that stigmatize and penalize women more severely than men, leading to underreporting and invisibility in addiction statistics (Greenfield et al., 2007; Amaro et al., 2001).

### 4.2 Psychological distress and ACEs as universal risk factors of substance use

Our findings reinforce the notion that psychological distress and ACEs are universal, key risk factors for adolescent substance use. Psychological distress had the strongest effect on both alcohol and drug use, indicating that adolescents experiencing higher levels of emotional distress are significantly more likely to engage in substance use. This aligns with previous research showing that substance use often serves as a coping mechanism for managing stress, anxiety, and depression in adolescents (McLaughlin et al., 2012; Chaiton et al., 2009). The fact that psychological distress consistently predicted substance use across all countries suggests that its impact transcends cultural differences, making it a universal risk factor.

Similarly, our findings show that ACEs, including family history of substance use, physical abuse, and psychological abuse, are strongly associated with higher substance use in adolescents. These results are consistent with previous studies highlighting childhood trauma as one of the most significant predictors of substance use initiation and escalation in adolescence (Felitti et al., 1998; Dube et al., 2003). Research has consistently shown that adolescents exposed to early-life adversity are at increased risk of substance use due to heightened emotional dysregulation, impaired stress-response systems, and increased impulsivity (Anda et al., 2006; Lee and Chen, 2017).

Although our study found some cultural variation in how physical and psychological abuse influenced drug use, the overall pattern suggests that the link between childhood trauma and substance use is largely culture independent. This aligns with findings from a large-scale meta-analysis (Schilling et al., 2007) which demonstrated that the impact of ACEs on adolescent substance use remains significant across diverse cultural and socioeconomic backgrounds. Additionally, a WHO international study found that the association between childhood adversity and substance use disorders is evident across high-, middle-, and low-income countries, further supporting the universality of ACEs as a risk factor (Kessler et al., 2010).

### 4.3 Personality traits as universal predictors of substance use

Our findings indicate that personality traits consistently predict adolescent substance use across cultures, reinforcing the universality of their influence on drinking and drug behaviors. Extraversion was associated with higher alcohol and drug use, while conscientiousness, agreeableness, openness, and neuroticism were protective factors against substance use. The lack of significant interaction effects between countries and personality traits suggests that these associations remain stable across diverse cultural contexts, indicating that personality-related risk and protective mechanisms for substance use are culture independent. These findings align with extensive research in adults demonstrating the cross-cultural stability of the Big Five personality traits in relation to substance use. A meta-analysis by Hakulinen et al. (2015), which included studies from multiple countries, found that extraversion was consistently linked to greater alcohol and drug use, while conscientiousness and agreeableness were protective across all examined cultures.

One possible explanation for this finding is that core personality traits shape individual behaviors through universal psychological mechanisms. Notably, extraverted individuals are more prone to sensation-seeking, impulsivity, and peer-driven behaviors, making them more likely to engage in risky substance use, regardless of cultural setting (Quinn and Harden, 2013). Conversely, conscientiousness is associated with self-control, long-term goal orientation, and risk aversion, providing a protective buffer against substance use across all societies (Bogg and Roberts, 2013). Additionally, studies have shown that personality traits influence substance use development early in life, often preceding cultural or environmental influences. Martínez-Gabaldón and Martínez-Pérez (2024) found that conscientiousness and agreeableness measured in childhood reliably predicted lower substance use in adulthood, suggesting that personality-based risk factors emerge before cultural and societal norms begin shaping behavior.

A central conclusion from this study is the remarkable cross-cultural stability of key predictors. Psychological distress, personality traits, and ACEs consistently predicted substance use regardless of cultural background, with only limited evidence for moderation effects. This suggests that while the prevalence and patterns of substance use vary by country, the underlying mechanisms linking individual risk factors to substance use are largely universal.

### 4.4 Strengths and limitations

A key strength of this study is its large and diverse sample, consisting of over 5,000 adolescents aged 15–19 from five culturally distinct countries (Sweden, Vietnam, Serbia, Morocco, and the United States). The simultaneous data collection across all countries enhances comparability and reduces the risk of temporal biases. Another strength is the unusually high number of responses obtained through an online survey platform, which contrasts with the typically lower response rates and higher levels of missing data often associated with online survey research (Wu et al., 2022). This suggests that the topic and study design were engaging and accessible to adolescents across diverse contexts. However, since the number of individuals exposed to the recruitment campaign is unknown, the true response rate cannot be determined, limiting conclusions about the campaign’s overall reach and effectiveness.

Additionally, the use of self-reported gender identity allows for a more inclusive and nuanced understanding of gender differences in substance use. The reliance on self-reported measures, while often seen as a limitation, can also be considered a strength, as it captures adolescents’ subjective experiences and perceptions of substance use, psychological distress, and ACEs in a way that objective clinical data might not fully reflect. Furthermore, the study assessed both alcohol use (AUDIT scores) and drug use (DUDIT scores) within the past 12 months, providing a comprehensive picture of substance use behaviors.

Despite these strengths, the study has several limitations. Self-report data are susceptible to biases, including underreporting or overreporting of substance use due to social desirability, recall issues, or fear of judgment. Additionally, substance use was defined to include only alcohol and drugs, excluding tobacco products, which are also commonly classified as psychoactive substances in public health research. Furthermore, potential unmeasured cultural factors, such as family structures, peer influence, or national differences in substance availability and policy enforcement, that may have influenced the results were not directly assessed.

Another limitation is the unequal distribution of participants across countries. Sweden had the largest proportion of respondents (31.4%), followed by Vietnam (30%), Serbia (21.7%), Morocco (10.6%), and the United States (6.3%). This imbalance may have affected the statistical power to detect cultural differences and limits the generalizability of findings, particularly for the United States and Morocco, which had small samples.

Additionally, data collection occurred during the COVID-19 pandemic, which may have influenced adolescents’ substance use behaviors and self-reports due to varying pandemic-related restrictions, school closures, and social limitations. Since the AUDIT and DUDIT assess substance use in the past twelve months, the pandemic’s impact on accessibility to substances, stress levels, and coping mechanisms may have influenced responses, adding a potential confounding factor.

### 4.5 Implications for prevention and intervention

These findings emphasize the critical need for trauma-informed and mental health-focused interventions in substance use prevention efforts. Given that psychological distress and ACEs increase substance use risk regardless of cultural background, prevention and intervention programs should prioritize early screening for childhood trauma and mental health struggles. Additionally, integrating these insights into evidence-based coping strategy treatment programs for adolescents, such as cognitive behavioral therapy (CBT) and resilience-building programs, could help mitigate the long-term impact of early-life adversity on adolescent substance use.

Given the consistent associations between personality traits and substance use across countries, intervention strategies targeting substance use among adolescents should incorporate personality-based prevention approaches. For example, adolescents with high extraversion and low conscientiousness may benefit from early interventions focused on impulse control, risk perception, and peer resistance skills. Programs using personality-targeted interventions, such as the Preventure Program (Conrod et al., 2010), have been shown to reduce substance use in high-risk adolescents by addressing their underlying personality traits rather than focusing solely on cultural or environmental influences.

### 4.6 Conclusion

Across five countries, cultural differences in prevalence coexist with a common set of drivers: psychological distress and early adversity elevate alcohol and drug use, whereas conscientiousness and agreeableness are protective and extraversion signals higher risk. These consistent patterns suggest that prevention can be both culturally sensitive and portable, combining trauma-informed mental-health supports with personality-targeted approaches for vulnerable youth. By clarifying which factors matter across settings, our findings help practitioners and policymakers prioritize interventions with the greatest potential for broad, real-world impact.

## Data Availability

The raw data supporting the conclusions of this article will be made available by the corresponding author upon reasonable request.

## Footnotes

1
https://meshe.se/
